# Donald Trump, Dominating Masculine Necropolitics, and Covid-19

**DOI:** 10.1177/1097184X20984816

**Published:** 2020-12-30

**Authors:** James W. Messerschmidt

**Affiliations:** 1 University of Southern Maine, Portland, ME, USA

In this short essay, I concentrate on US president, Donald J. Trump, and what I label his “dominating masculine necropolitics.” *Dominating masculinity* involves commanding and controlling specific interactions, and exercising power and control over people and events—“calling the shots” and “running the show” (Messerschmidt 2016). Differing from *hegemonic masculinities,* dominating masculinities do not necessarily legitimate a hierarchical relationship between men and women, masculinity and femininity, and among masculinities. In that sense, then, dominating masculinities are often, but not always, analytically distinct from hegemonic masculinities (see Messerschmidt 2016, 2018).

Trump’s specific form of dominating masculinity involved commanding and controlling specific interactions, and exercising power and control over people and events; he called the shots and ran the show, demanded strict obedience to his authority, and displayed a lack of concern for the opinions of others. Throughout Trump’s presidency, this dominating masculinity centered on several critical features, which were emphasized or de-emphasized depending upon the context. For example, Trump cultivated control over close followers based on loyalty to him as a person rather than to a political party or set of principles; he adapted the office of the presidency to serve his needs rather than submit to shared custom; he asserted sweeping executive powers while subverting congressional oversight; he controlled public discourse through his constant tweets; he formulated a dominating militaristic foreign policy; and he became a functioning member of a global ultraconservative “axis of evil” whose defining characteristics were kleptocracy and dominating masculinity (Messerschmidt 2019; Messerschmidt and Bridges 2018).

The arrival and spread of Covid-19 around the world provided a new and dangerous context within which Trump’s dominating masculinity has been increasingly constructed through novel necropolitical practices. Achille Mbembe (2003, 2019) defines “necropolitics” as the use of social and political power to determine who may live and who may die, and who is disposable and expendable. Trump’s “policy” on Covid-19, or lack thereof, was a profound example of *dominating masculine necropolitics*. In this essay, I describe how this materialized.

The first case of Covid-19 recognized in the United States was on January 20, 2020. Throughout January and February, and into March of that year, Trump was continually warned by medical experts of the health threat and advised to take the virus seriously (Lipton et al. 2020). In line with his ongoing dominating masculinity, he dismissed the advice of his medical and scientific advisers by responding that they should not “panic” or be “alarmist.” Publicly, he repeatedly downplayed the severity of the virus. His medical experts urged a national policy of physical distancing; testing, tracing, and isolation; and the provision of personal protective equipment (Lipton et al. 2020). Instead of implementing such a domestic policy, Trump decided he alone would be calling the shots, and he dominated public discourse on the virus. For example, on January 22, Trump brazenly declared that *he* had the virus “totally under control.”

Although Trump’s medical experts continued to advocate for mitigation in order to minimize the spread of the virus, Trump unwaveringly stayed on message by continually downplaying the danger of the virus, claiming that “we’re very cognizant of everything going on. We have it very much under control in this country” (February 23). By February 25, it was reported that there were 15 known cases of Covid-19 in the United States, and on that day, Dr Nancy Messonnier, director of the National Center for Immunization and Respiratory Diseases at the Centers for Disease Control and Prevention (CDC) and a member of the White House Coronavirus Task Force, challenged Trump’s control over Covid-19 discourse by stating at a press briefing: “We expect we will see community spread in this country. It’s not so much of a question of if this [outbreak] will happen anymore but rather more of a question of exactly when this will happen and how many people in this country will have severe illness.” Immediately following these comments, Messonnier was silenced by Trump, never appearing again at public briefings by the task force, and this subordination is one illustration of his demand for strict obedience to his authority. On the next day (February 26), Trump stated that an outbreak was not “inevitable,” and in fact “within a couple of days it is going to be down close to zero.” The following day he insisted “it’s going to disappear. One day it’s like a miracle, it will disappear” (February 27).

What this suggests is that Trump exercised power and control over the pandemic message and tactics while simultaneously displaying a lack of concern for the opinion of his medical experts. Trump decided that he alone would solve the pandemic as he claimed to have a “natural ability” at medicine, and thus he attempted to flaunt his self-appointed medical adeptness by wondering out loud about the curative potential of injecting disinfectant and the antimalarial drug hydroxychloroquine (Yong 2020). People quickly understood the absurdity of injecting disinfectant, and studies predictably revealed that hydroxychloroquine was ineffective (Case Western Reserve University 2020; National Institutes of Health 2020).

The first Covid-19 death in the United States occurred on March 1, 2020. Yet Trump did not assert a national emergency until 13 days later (Perano 2020). But by this time the virus had durably established itself in the United States and began to spread throughout the population. By early April, the United States recorded 2,850 Covid-19 deaths, and by May the number reached 55,337. A study was published online in late May, concluding that the United States could have prevented 62 percent of reported infections and 55 percent of reported deaths if Trump had developed a national strategy of mitigation just one–two weeks earlier (Pei, Kandula, and Shaman 2020). Yet Trump’s national emergency did not involve a national strategy of mitigation. Instead, Trump’s response on May 19 was: “When we have a lot of cases, I don’t look at that as a bad thing, I look at that as being a good thing. It means our testing is much better. I view it as a badge of honor.” By June, the number of Covid-19 deaths had increased to 102,640, by August it was at 151,265, it exceeded 200,000 by October, in November it surpassed 250,000, and during December it eclipsed 300,000, uniformly killing between one and two people *every minute*; thus it was ranked as *the leading cause of death* in the United States. Yet, Trump claimed success because Covid-19 had not killed 2.2 million US citizens.

Trump’s dominating masculine necropolitics especially involved flouting guidelines for mask wearing. Trump chose from the beginning not to wear a mask during most public appearances. Following the CDC suggestion on April 3 that people wear masks in public, Trump announced that masking actually is optional: “You don’t have to do it. I’m choosing not to do it.” Trump’s motive for not wearing a mask centered on his concern of looking “ridiculous” and “weak” (Associated Press 2020). And Trump’s disparaging of mask wearing quickly became a masculine loyalty test, particularly among men, who were less likely than women to wear a mask because it feels “shameful,” “not cool,” and “[is] a sign of weakness” (Jagannathan 2020; Mahdawi 2020).

During the final weeks of the presidential campaign, Trump held super-spreader political rallies—that were followed by community virus outbreaks—in which very few attendees wore masks and practiced physical distancing (Nayer 2020). One study examined the aftereffects of 18 Trump rallies from June 20 to September 30, concluding a high likelihood that the rallies led to over 30,000 extra Covid-19 cases and over 700 deaths that otherwise would have been avoided (Bernheim et al. 2020). Evidence suggested that coronavirus hospitalizations increased dramatically in counties without a local mask mandate compared to counties with a mask directive, and if 95 percent of the US population regularly wore masks in public, 130,000 lives would be saved (Graves 2020; Reiner 2020). Trump also used his own infection and hospitalization to double down on his dominating masculine necropolitics by proclaiming to the country after treatment with experimental drugs: “Don’t be afraid of Covid” and “don’t let it dominate you.”

Trump’s dominating masculine necropolitics during Covid-19 worked to structure some people’s lives as more expendable than others, resulting in tens of thousands of avoidable deaths, particularly among Black Americans who died disproportionately of Covid-19 when compared with the rest of the US population (Williams 2020). According to Sarah Anderson and Brian Wakamo (2020), Black Americans were more than twice as likely to die from Covid-19 than White Americans, and as of September 15, 2020, for every 100,000 people, approximately 98 Black Americans and 47 White Americans died from the virus. These authors also pointed out that the pandemic-related economic crisis was disproportionately devasting for Black Americans, whose unemployment rate in September was 12.1 percent compared to 7.0 percent for White Americans. The higher risk of contracting coronavirus and dying among Black Americans is due to larger racial and class inequalities: access to health care, reliance on public transportation, likelihood of employment in positions that augment exposure to the virus, racial segregation in more densely populated areas, and substandard treatment by health care providers (McCoy 2020). Black Americans constitute 13 percent of the US population, but they suffered 23 percent of all Covid-19 deaths, and more than 80 percent of Covid-19 deaths occur in people aged 65 years and over (Berezow, 2020). Consequently, Trump’s dominating masculine necropolitics exposes particular US citizens to a greater likelihood of death: Black Americans, lower- and working-class people, and the elderly have been designated disposable and expendable.

The culprit for the staggering spread of Covid-19 and premature death within the United States is Trump and his dominating masculine necropolitical discourse and practice. It is Trump who exercised power and control over this epidemic in the United States (until January 20, 2021). He called the shots; demanded strict adherence to his authority; flaunted a racist, classist, and ageist contempt for human life; and displayed a lack of concern for the opinions of medical experts and studies. Under Trump’s rule, the United States accounted for one-fifth of all Covid-19 deaths worldwide. The failure of Trump to construct a caring masculinity (Elliott 2016) that recognizes the horrors of this virus, sympathizes with those infected, respects all human life, and involves a meticulous national strategy for testing, tracing, and isolating as well as masking and maintaining physical distance resulted in somewhere between 130,000 and 210,000 excess US citizen deaths (Redlener et al. 2020). Indeed, Covid-19 mortality rates in countries that practiced a *non-dominating* feminine or masculine approach—such as New Zealand (Wade and Bridges, 2020) and South Korea (Redlener et al., 2020)—indicated that the extent of death reached in the United States under Trump was entirely avoidable.
